# Protein structural domain-disease association prediction based on heterogeneous networks

**DOI:** 10.1186/s12864-024-11117-0

**Published:** 2025-04-10

**Authors:** Jingpu Zhang, Lianping Deng, Lei Deng

**Affiliations:** 1 School of Computer and Data Science, Henan University of Urban Construction, 467000 Pingdingshan, China; 2 School of Computer Science and Engineering, Central South University, 410075 Changsha, China

**Keywords:** Heterogeneous networks, Meta-path topological feature, Domain-disease association prediction

## Abstract

**Background:**

Domains can be viewed as portable units of protein structure, folding, function, evolution, and design. Small proteins are often found to be composed of only a single domain, while most large proteins consist of multiple domains for achieving various composite cellular functions. A dysfunction in domains may affect the function of proteins in some disease. Inferring the disease-related domains will help our understanding of the mechanism of human complex diseases.

**Results:**

In this study, we firstly build a global heterogeneous information network based on structural-based domains, proteins, and diseases. Then the topological features of the network are extracted according to the meta-paths between domain and disease nodes. Finally, we train a binary classifier based on the XGBOOST (eXtreme Gradient Boosting) algorithm to predict the potential associations between domains and diseases. The results show that the binary classification model using the XGBOOST algorithm performs significantly better than models using other machine learning algorithms, achieving an AUC (Area Under Curve) score of 0.94 in the leave-one-out cross-validation experiment.

**Conclusions:**

We develop a method to build a binary classifier using the topological features based on meta-paths and predict the potential associations between domains and diseases. Based on its predictive performance in independent test sets, the method is proved to be powerful. Moreover, representing domains and diseases through integrating more multi-omic data will further optimize predictive performance.

**Supplementary Information:**

The online version contains supplementary material available at 10.1186/s12864-024-11117-0.

## Background

Modern human and medical genetics have been revolutionizing the use of gene-mapping techniques (such as linkage analysis and association studies) to search for genetic variations in complex human diseases, which has set off a wave of biomedical research [1, 2]. For example, the Human Genome Project and the HapMap Project, combined with GWAS and sequencing studies, have supported identification of more than 60,000 genetic associations across thousands of human diseases and traits [3]. Even if the particular disease associated genes are identified [4–8], narrowing it down to a specific structure-based protein domain is very challenging because genes control the proteins containing single or multiple domains [9, 10]. A protein domain is a conserved and functional unit of a protein, which can form a relatively independent spatial structure by folding, and each structure-based domain has a unique function [11]. Most proteins consist of one or several domains, and an identical domain may find in a variety of different proteins that capture specific functions [12, 13]. If a gene encodes a protein that contains a great deal of domains and is associated with a disease, one of the domains belonging to the protein may be related to the disease. Narrowing down domains related to complex diseases of human will greatly improve our understanding of complex diseases, and promote drug discovery and personalized treatment of complex diseases [14–16].

Domains fall into two categories, namely, sequence-based domains and structure-based domains. Sequence-based domains are usually obtained from the given protein sequence based on homology alone and the relations between human proteins and sequence-based domains are conveniently obtained from the Pfam database [17–20]. For this reason, there have been some researches on the potential associations between the sequence-based domains and diseases. For example, Wang et al. researched the relationships between human diseases and domains by using the principle of ’guilt-by-proximity’ [20]. Zhang et al. developed the method named domainRBF to infer the domains and human disease associations [21]. However, the structural-based domains are more convincing than the sequence-based domains in identifying protein functions [22, 23]. Thus, in this work, we research the structural-based domains related to diseases. As far as we know, there are no research to predict the relationships between structure-based domains and diseases at present.

In this article, we build heterogeneous information network containing domain-protein associations, disease similarities, domain-disease associations, protein-disease associations, and protein interactions. Inferring the associations between the domains and diseases can be transformed into the task of link prediction between the nodes in the heterogeneous information network. Unlike traditional homogeneous networks, heterogeneous information networks have many types of nodes, which can be connected through different relationships [24].

In order to obtain the topological characteristics in the heterogeneous information network to predict the potential associations between the structural-based domains of protein and diseases, we draw lessons from meta-path proposed by Sun et al [25]. The definition of a meta-path links a certain type of path of the start node and the end node. Each meta-path has a specific semantics. The sum of paths belonging to a particular meta-path is an important topological function for evaluating the strength of the association between the start node and the end node. For example, there are multiple paths between a domain and a disease: (A) domain $\rightarrow$ protein $\rightarrow$ protein $\rightarrow$ disease, and (B) domain $\rightarrow$ protein $\rightarrow$ disease $\rightarrow$ disease. The two meta-paths which connect the starting protein structural domain to the ending disease have different semantics: (A) if the protein is involved in a disease, the domain belonging to another protein interacting with the protein might be associated with the disease; (B) if a protein is associated with a disease, the domain in the protein might be related to other diseases similar to the disease.

We extract the topological features of the global network by designing different meta-paths. According to the topological features, we build binary classifier based on the XGBOOST (eXtreme Gradient Boosting) algorithm to predict the potential associations between structure-based domains and diseases.

## Materials and methods

### Datasets

To build the heterogeneous information network, we collected information about structure-based domains, proteins and diseases from different data sources. The data set mainly includes a disease similarity network data set, a structure-based domain and protein association network data set, a protein interaction network data set, a protein and disease association network data set and a structure-based domain and disease association network data set.

#### Domain-protein network

We downloaded structure based domains-protein associations from the SDADB database [12]. SDADB is a database of predicted annotations using the structural (SCOP) domain of the integrated method. We uniformly use the protein Uniport_ID as the ID that uniquely identifies the protein, and convert the PDB_ID in the SDADB database to the Uniport_ID through the relevant mapping file. Finally, after the data is processed, we finally obtain 1,756,074 domain-protein associations involving 140,189 domains and 35,866 proteins.

#### Protein-protein network

We collected protein interaction data sets from the STRING database [26]. Since this article only studies the association between human-related diseases and protein domains, we have selected only human protein interaction data sets. At present, many experiments have proved that combined_score$\ge$ 400 of the protein interaction is related, so we only select the associated data of combined_score$\ge$400. Secondly, we uniformly use the protein Uniport_ID as the ID that uniquely identifies the protein, and convert the String_ID in the STRING database to the Uniport_ID through the relevant mapping file. After de-redundancy, we collect 1,462,673 human protein interactions data set between 35,866 proteins from the STRING database.

#### Protein-disease network and disease-disease network

We obtained human-related disease phenotypic data from the OMIM database [27]. In this context, the disease phenotype is simply referred to as a disease. OMIM is one of the tools commonly used by genetic workers. It is a continuously updated database of human genes and disorders of genetic [28, 29]. It centered on heritable or disease about hereditary genetic. After data processing, we finally obtain 5,802 protein-disease associations between these 35,866 proteins and 5,099 diseases. Moreover, we download 88,665 phenotypic similarities about 5,099 diseases from a recent work of Reyes-Palomares et al [30].

#### Disease-domain network

To train and evaluate our classifier, we need to build the reference data set, namely the structural-based domain disease associations. However, there is no manually assigned structural-based domain-disease association data yet. Alternatively, we can obtain the association data by transferring the associations involving the single-domain proteins to their component domains, since the relationship between a single-domain protein and a disease is considered that it occurs between the domain generated from the protein and the disease. After data processing, we finally obtain 666 domain-disease associations between 140,189 domains and 5,099 diseases.

### Building a global heterogeneous information network

**Fig. 1:**
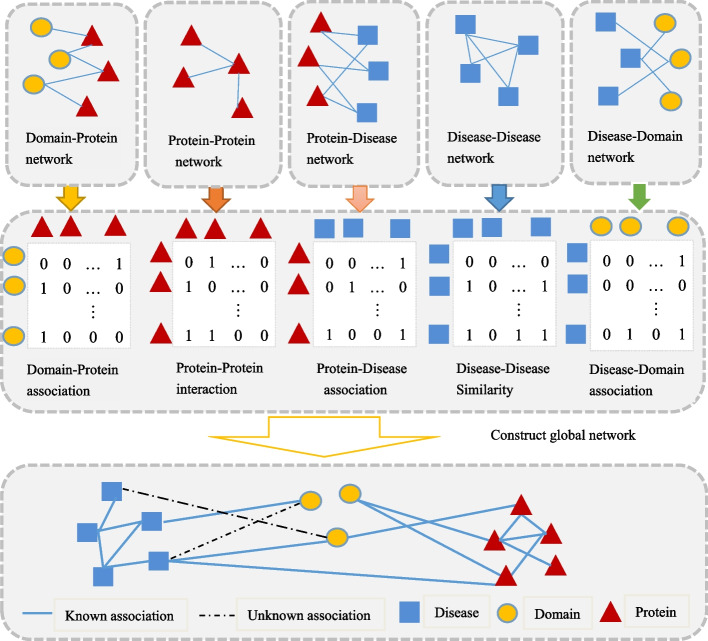
Five networks are firstly constructed according to the downloaded data. Then a global heterogeneous network, which is composed of five networks, is built

According to the data sources described above, we build a global heterogeneous network composed of domain-protein association network, protein interaction network, protein-disease association network, disease similarity network, domain-disease association network. The global heterogeneous network is denoted as G=(H, F), where H = D$\cup$P$\cup$S, D, P and S are the sets of structure-based domain, protein, disease nodes in the network respectively, while $\text {F} = \text {F}_{d,p}\cup \text {F}_{p,p} \cup \text {F}_{s,d} \cup \text {F}_{p,s} \cup \text {F}_{s,s} \cup \text {F}_{d,s} \cup \text {F}_{p,d} \cup \text {F}_{s,p}$ are the sets of heterogeneous links in G [31]. Based on the semantic links between any two nodes, a total of five adjacency matrices are calculated. The elements of the adjacency matrix have two values: ‘0’ for the unseen link and ‘1’ for the observed link. It is worth noting that these adjacency matrices are reversible, and the inverse association matrices can be derived from the transposition of the original matrices. The global heterogeneous information network is shown in Fig. 1.

### Extracting meta-path-based topological features

**Fig. 2:**
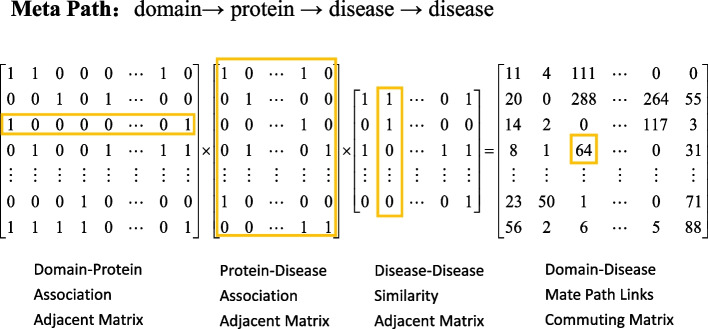
Schematic representation of calculations of the commuting matrix through multiplying three adjacency matrices

As described above, the total number of paths belonging to a particular meta-path is an important topological feature that can be extracted from a heterogeneous network to assess the strength of the association between the start and end nodes. Hence, we construct the meta-paths to extract topological features of domain and disease nodes in the network [32]. The meta-path topological features are encoded in commuting matrices. Each commuting matrix represents a certain type of meta-path of a given length. Each element in the commuting matrix represents the number of path instances that link one domain to one disease, and the value in the matrix is non-negative. Figure 2 takes one of the meta-paths (e.g. domain $\rightarrow$ protein $\rightarrow$ disease $\rightarrow$ disease) to demonstrate the calculation of commuting matrix. The commuting matrix in the example is calculated by multiplying domain-protein association adjacency matrix, protein-disease association adjacency matrix and disease-disease similarity adjacency matrix [25, 33].

To predict the potential associations between structural-based domains and disease, we list all possible meta-paths, producing a total of 27 meta-paths. The length of the meta-path is equal to the number of adjacency matrices multiplied. Among the 27 commuter matrices, there are 2 meta-paths with step size 2, there are 4 meta-paths with step size 3, there are 11 meta-paths with step size 4, there are 10 meta-paths with step size 5. All meta-paths are as shown in Table 1.

**Table 1:** The meta-paths from domains to diseases in the heterogeneous network

Name	Pathway scheme	Pathway
C1	DPS	domain$\rightarrow$ protein $\rightarrow$ disease
C2	DSS	domain$\rightarrow$ disease $\rightarrow$ disease
C3	DPSS	domain$\rightarrow$ protein $\rightarrow$ disease $\rightarrow$ disease
C4	DPPS	domain$\rightarrow$ protein $\rightarrow$ protein $\rightarrow$ disease
C5	DSPS	domain$\rightarrow$ disease $\rightarrow$ protein $\rightarrow$ disease
C6	DPDS	domain$\rightarrow$ protein $\rightarrow$ domain $\rightarrow$ disease
C7	DPPSS	domain$\rightarrow$ protein $\rightarrow$ protein $\rightarrow$ disease $\rightarrow$ disease
C8	DPSDS	domain$\rightarrow$ protein $\rightarrow$ disease $\rightarrow$ domain $\rightarrow$ disease
C9	DPDSS	domain$\rightarrow$ protein $\rightarrow$ domain $\rightarrow$ disease $\rightarrow$ disease
C10	DPSPS	domain$\rightarrow$ protein $\rightarrow$ disease $\rightarrow$ protein $\rightarrow$ disease
C11	DSPPS	domain$\rightarrow$ disease $\rightarrow$ protein $\rightarrow$ protein $\rightarrow$ disease
C12	DSPSS	domain$\rightarrow$ disease $\rightarrow$ protein $\rightarrow$ disease $\rightarrow$ disease
C13	DSDPS	domain$\rightarrow$ disease $\rightarrow$ domain $\rightarrow$ protein $\rightarrow$ disease
C14	DSPDS	domain$\rightarrow$ disease $\rightarrow$ protein $\rightarrow$ domain $\rightarrow$ disease
C15	DSSDS	domain$\rightarrow$ disease $\rightarrow$ disease $\rightarrow$ domain $\rightarrow$ disease
C16	DPPDS	domain$\rightarrow$ protein $\rightarrow$ protein $\rightarrow$ domain $\rightarrow$ disease
C17	DSSPS	domain$\rightarrow$ disease $\rightarrow$ disease $\rightarrow$ protein $\rightarrow$ disease
C18	DPPDSS	domain$\rightarrow$ protein $\rightarrow$ protein $\rightarrow$ domain $\rightarrow$ disease $\rightarrow$ disease
C19	DSSPPS	domain$\rightarrow$ disease $\rightarrow$ disease $\rightarrow$ protein $\rightarrow$ protein $\rightarrow$ disease
C20	DPPSDS	domain$\rightarrow$ protein $\rightarrow$ protein $\rightarrow$ disease $\rightarrow$ domain $\rightarrow$ disease
C21	DSSPDS	domain$\rightarrow$ disease $\rightarrow$ disease $\rightarrow$ protein $\rightarrow$ domain $\rightarrow$ disease
C22	DPDSPS	domain$\rightarrow$ protein $\rightarrow$ domain $\rightarrow$ disease $\rightarrow$ protein $\rightarrow$ disease
C23	DPPSPS	domain$\rightarrow$ protein $\rightarrow$ protein $\rightarrow$ disease $\rightarrow$ protein $\rightarrow$ disease
C24	DSDPDS	domain$\rightarrow$ disease $\rightarrow$ domain $\rightarrow$ protein $\rightarrow$ domain $\rightarrow$ disease
C25	DPSSDS	domain$\rightarrow$ protein $\rightarrow$ disease $\rightarrow$ disease $\rightarrow$ domain $\rightarrow$ disease
C26	DPSPPS	domain$\rightarrow$ protein $\rightarrow$ disease $\rightarrow$ protein $\rightarrow$ protein $\rightarrow$ disease
C27	DSPDSS	domain$\rightarrow$ disease $\rightarrow$ protein $\rightarrow$ domain $\rightarrow$ disease $\rightarrow$ disease

Each element in the commuting matrix is denoted by $M_{i,j}$, which indicates the number of path instances between nodes *i* and *j*. We normalize the number of path instances by running Random Walk (RW) algorithm. RW is computed as $M_{i,j} / M_{i}$, where $M_i$ is row-wise summations.

### Binary classification model based on XGBOOST algorithm

XGBOOST is an optimized version of the Gradient Boosting algorithm. It is developed for the purpose of speed and performance. Gradient boosting is an algorithm in which new models are created to predict the residuals of prior models and then add together to make the final prediction. This approach supports both regression and classification. In the manuscript, XGBOOST is employed to predict the potential associations between domains and diseases.

In order to train a XGBOOST classifier model, the positive and negative samples need to be obtained. We regard a total of 666 domain-disease associations downloaded as positive samples, and then randomly combine those unrelated data at a ratio of 1:1 as negative samples. In order to accurately evaluate the predictive performance without prior knowledge, the links we removed from the heterogeneous information network are used as positive label data in the test set. It is then combined with the negative label data as a test set. Training set accounts for two-thirds of the total data set, and test set accounts for one-third of the total data set. Therefore, in the training set, the total number of training sets is 888 link data set, including 444 link data set marked positive and 444 link data set marked negative. In the test set, the total number of training set are 444 link data set, there are including 222 positively labeled link data set and 222 negatively labeled link data set [25].

### Evaluation measures

To evaluate the prediction performances of the classifier, we employ two metrics, which are the area under the ROC curve (AUC), $F_1$ score respectively. For different classification thresholds, ROC curve graphs were drawn according to a relational function of true positive rate (TPR) and false positive rate (FPR). For each threshold, the corresponding TPR and FPR are computed. The ROC curve is obtained by changing the threshold [34]. The AUC to measure the overall performance is calculated.

$F_1$ score is an indicator used to assess the accuracy of binary classification prediction model, which can be thought of as a harmonic average of the model accuracy rate and the recall rate. It takes into account the accuracy and recall of the classification model. The definition is given by:

1 $$\begin{aligned} F_1 = \frac{ 2TP }{ 2TP + FN + FP } \end{aligned}$$

## Results

### XGBOOST model

To achieve the optimal prediction performance, it is necessary to tune the parameters in XGBOOST algorithm carefully. However, adjusting parameters can be a very difficult task because it has many parameters. In the work, the optimal values for tuning parameters, namely n_estimators, max_depth, min_child_weight, gamma, colsample_bytree, reg_alpha are tuned through the grid search. Other parameters are set to the default values. The performance of the XGBOOST model is evaluated by 3-fold cross validation. First of all, the validation data set is randomly divided into three groups of equal size, one group is used as the test set, and the other two groups are used as the training set. The experiment is repeated three times so that each set is hidden once and each hidden domain-disease pair is used to testing the performance of the model. Then take the average value as the final performance evaluation result.

### Effect of RW normalization on performance

**Fig. 3:**
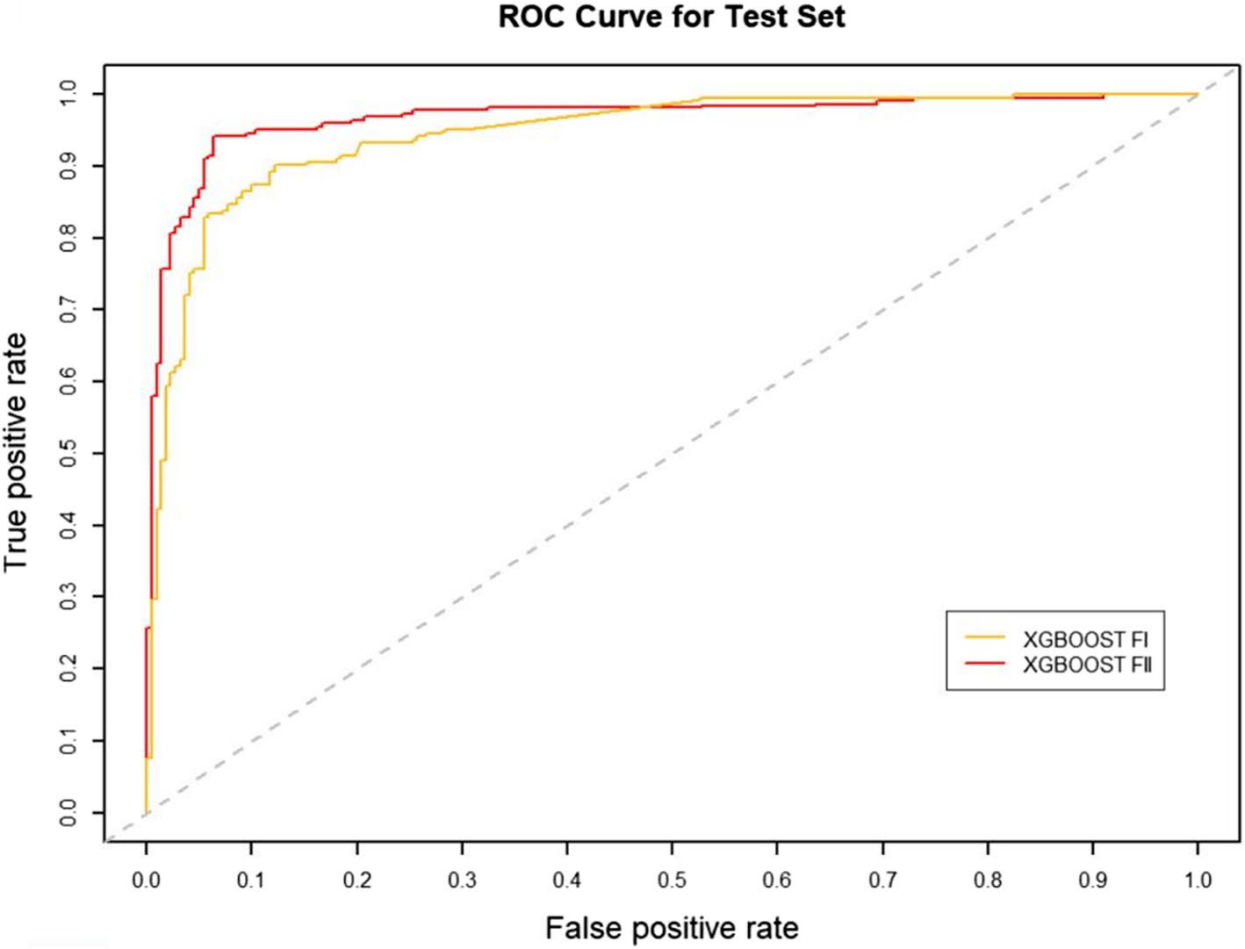
The AUC comparison of XGBOOST between feature set I and feature set II

**Fig. 4:**
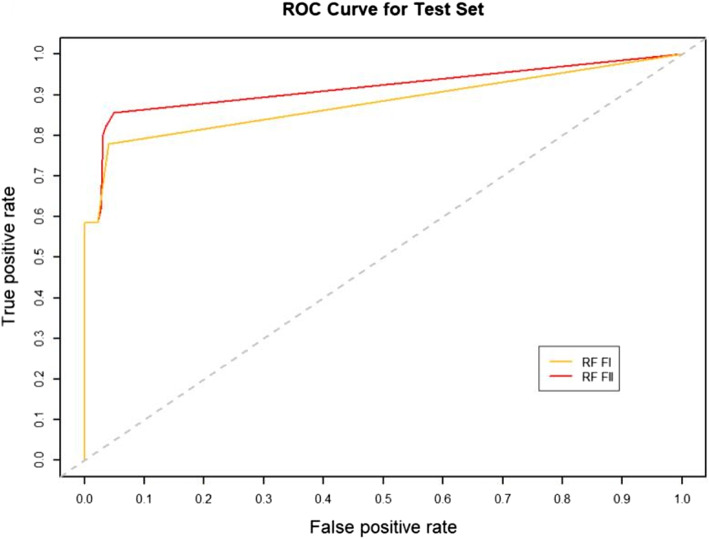
The AUC comparison of RF between feature set I and feature set II

**Fig. 5:**
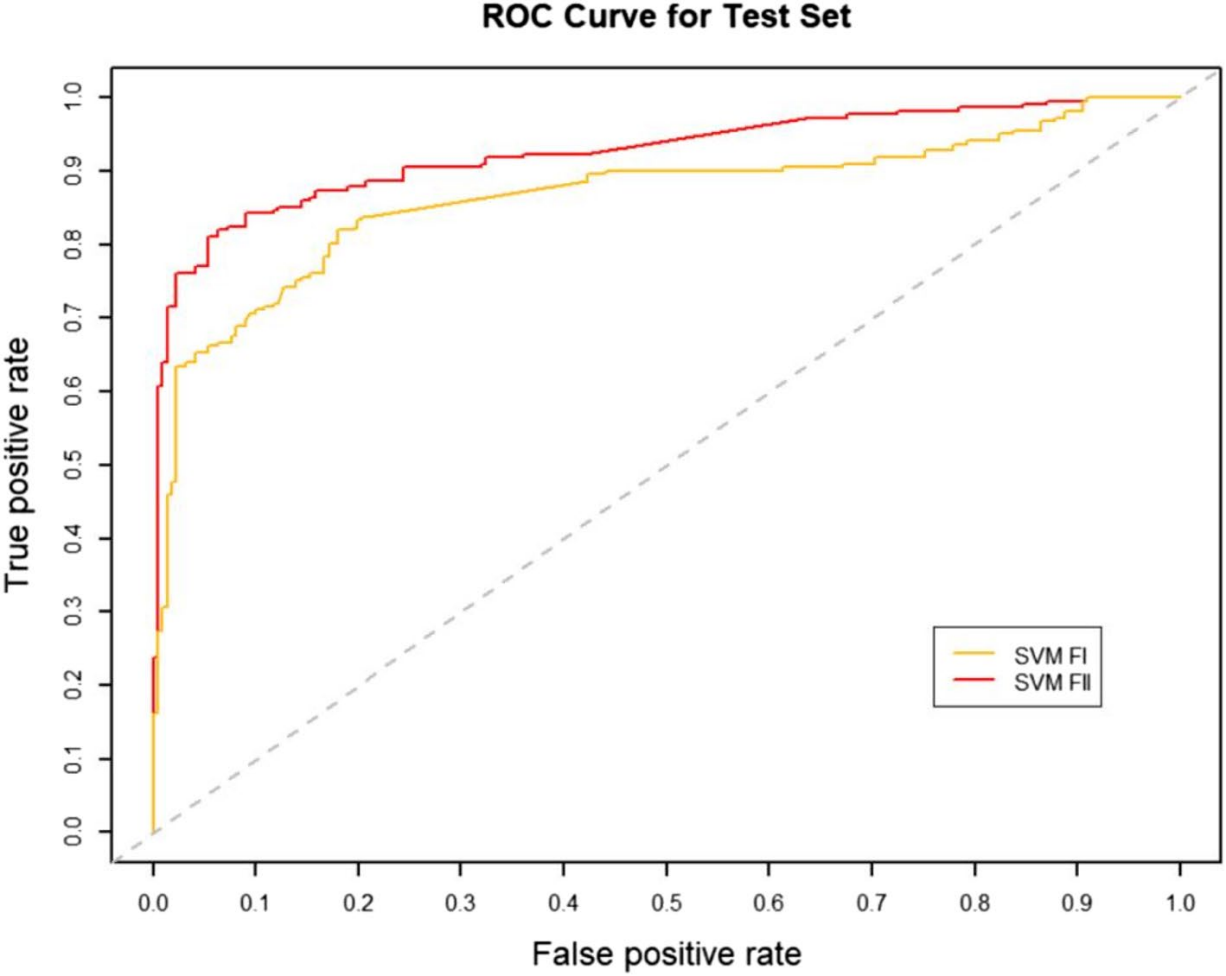
The AUC comparison of SVM between feature set I and feature set II

**Fig. 6:**
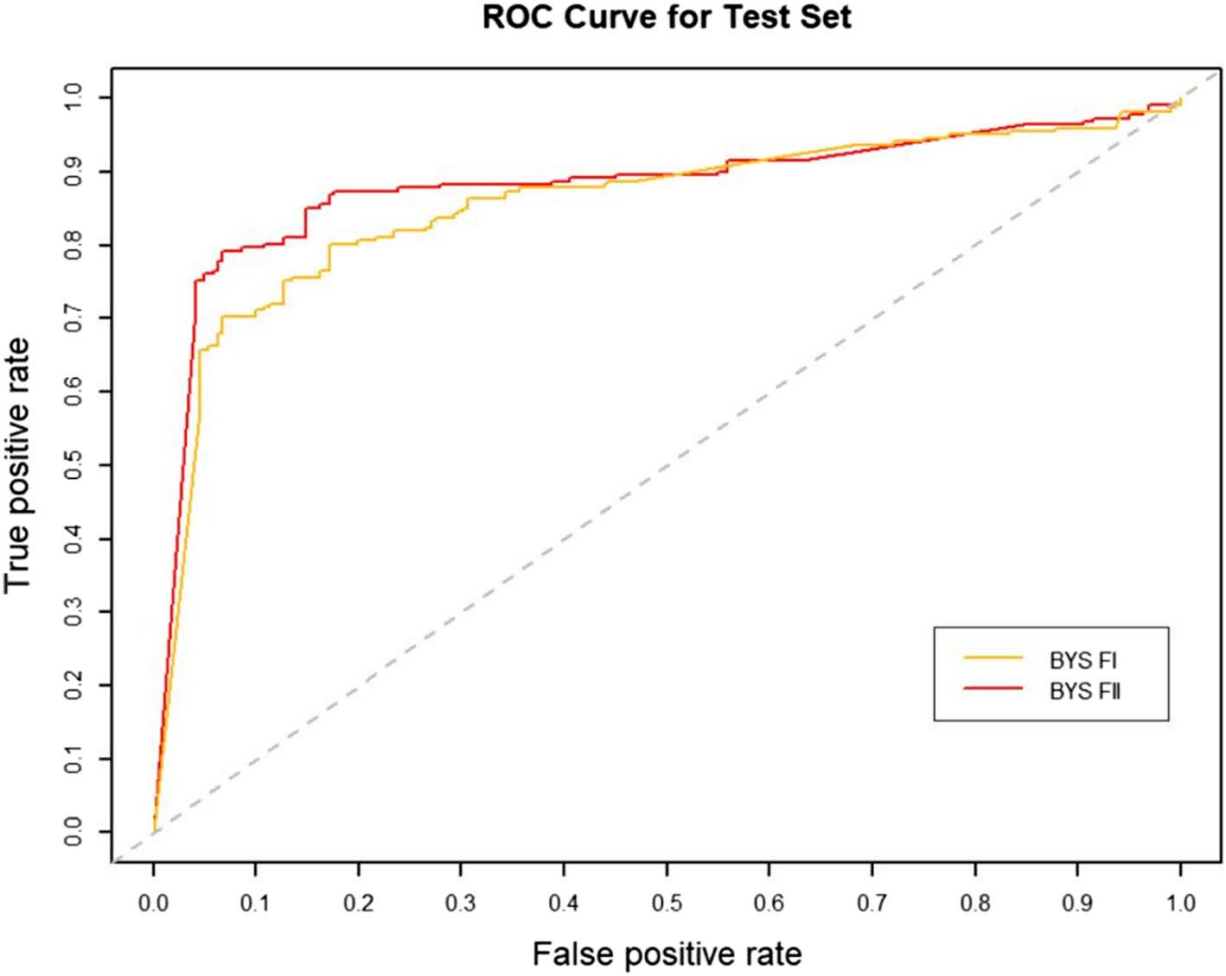
The AUC comparison of BYS between feature set I and feature set II

**Fig. 7:**
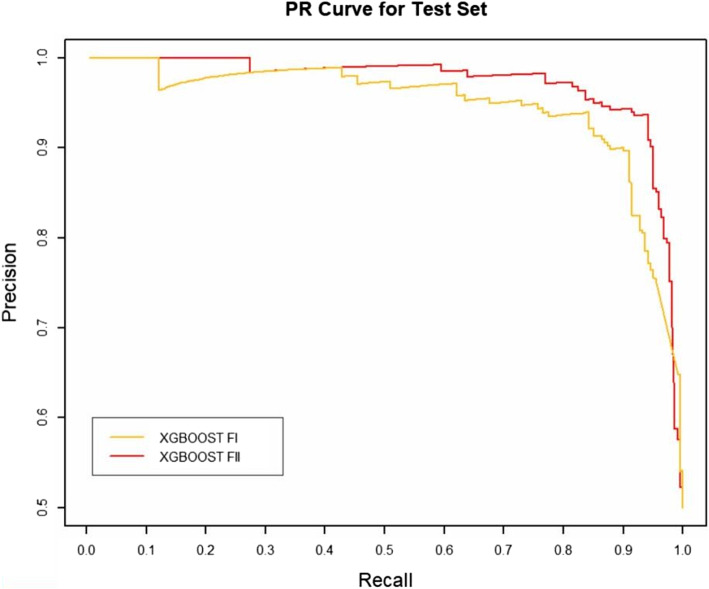
The PR comparison of XGBOOST between feature set I and feature set II

**Fig. 8:**
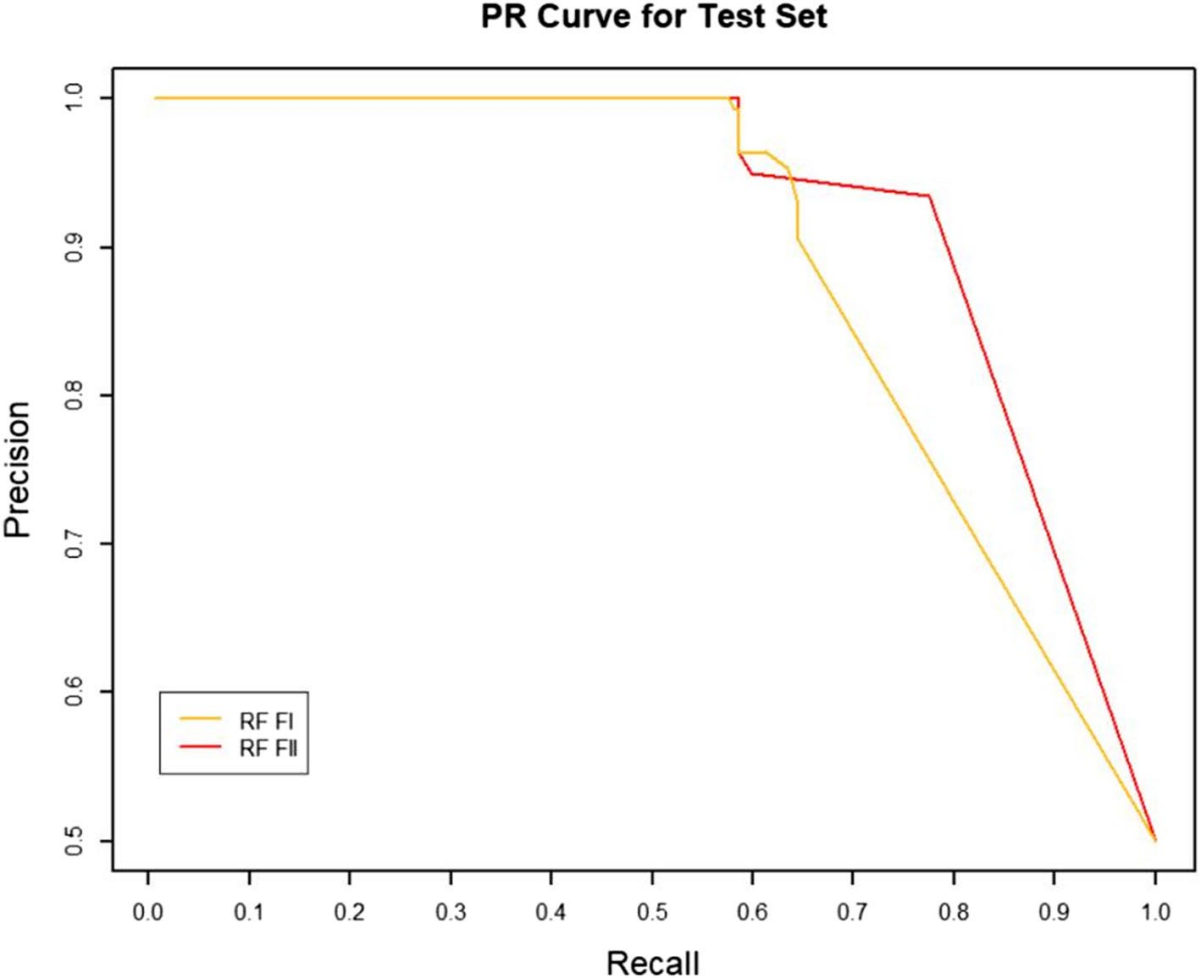
The PR comparison of RF between feature set I and feature set II

**Fig. 9:**
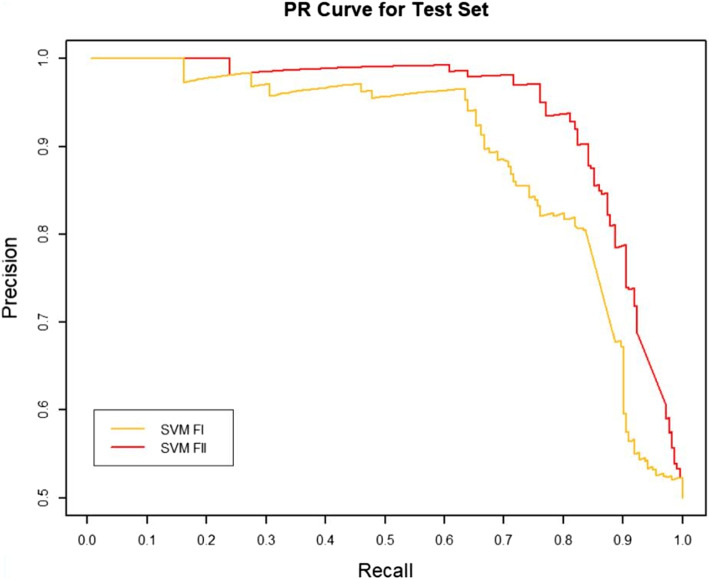
The PR comparison of SVM between feature set I and feature set II

**Fig. 10:**
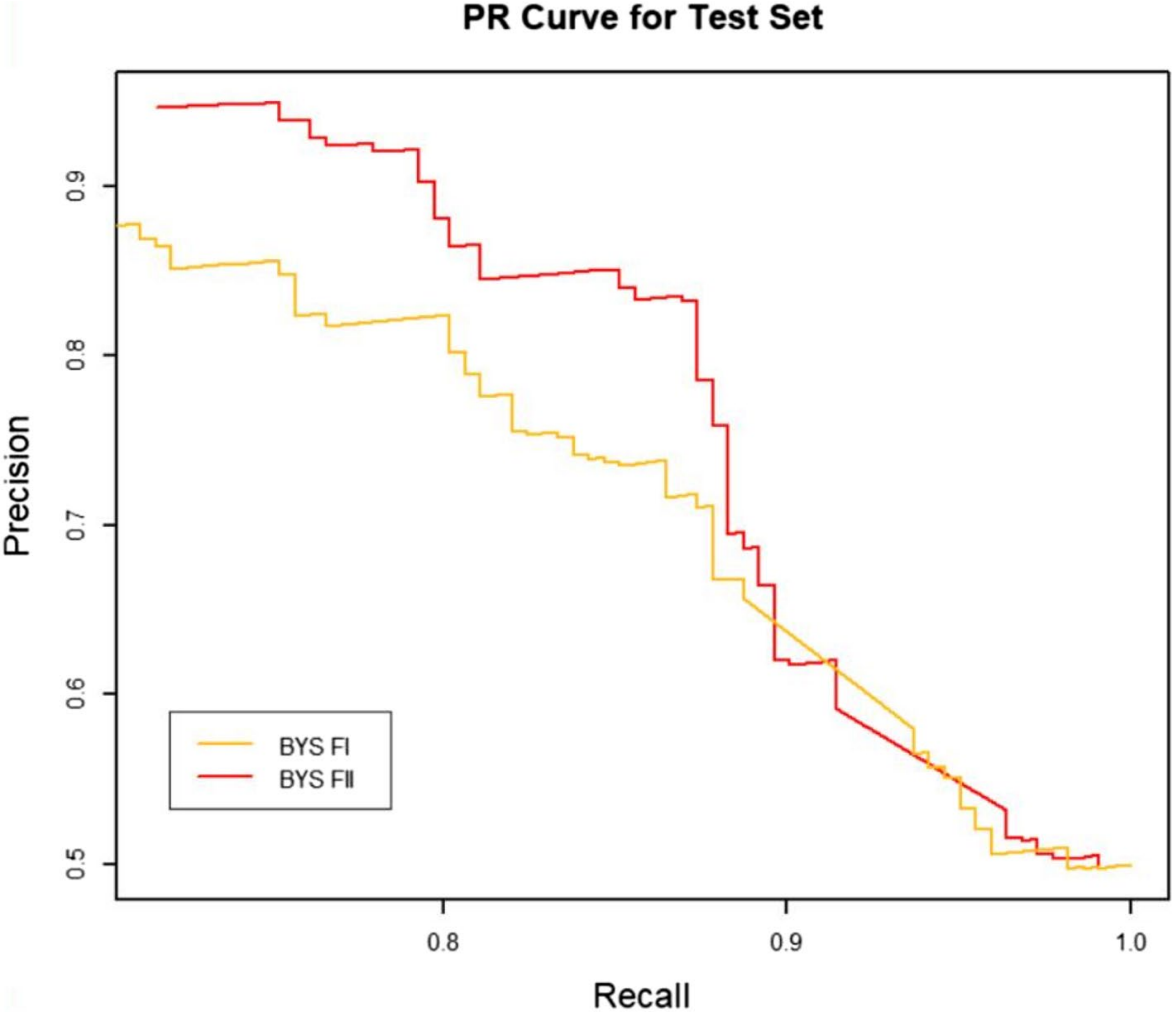
The PR comparison of BYS between feature set I and feature set II

RW normalization [35] of the features will make it possible to improve the predictive performance of the binary classifier. To verify this, we constructed two sets of topological features according to the path count. Feature-I contains the total number of all paths encoded by 27 commuter matrices, which are provided in the Additional files 1 and 3, respectively. The RW was normalized to the topological characteristics of the total number of paths for the 27 commuter matrices, and by combining the path count and RW normalization, we obtained Feature-II containing 54 topological characteristics, which are provided in the Additional files 2 and 4, respectively. We run four classifiers (XGBOOST (eXtreme Gradient Boosting), RF (Random Forest), SVM (Support Vector Machine) and BYS (Naive Bayes)) on Feature-I and Feature-II respectively. The performance comparisons of the four classifiers between the two topological feature sets are shown in Table 2 and Figs. 3, 4, 5, 6, 7, 8, 9, and 10.

**Table 2:** Performance comparison based on two topological feature sets of among different models

Methods	Topological features	AUC	*F*_1_ scores
XGBOOST	Feature-I	0.9341	0.8823
	Feature-II	0.9461	0.8942
RF	Feature-I	0.8791	0.8564
	Feature-II	0.9280	0.8738
SVM	Feature-I	0.8658	0.8213
	Feature-II	0.9255	0.8718
BYS	Feature-I	0.8554	0.8127
	Feature-II	0.8805	0.8527

From the table and these figures, we can find the ranking of the predictive performance of all the models on two different feature sets: Feature-II > Feature-I. The predictive performance of XGBOOST model is improved by about 1%, 1% in terms of AUC and $F_1$ score respectively. Besides XGBOOST, the other three models also have improvements in performances in AUC and $F_1$ score. The results demonstrate that RW normalization can greatly improve the predictive performance by enriching the feature space.

### Influence of the length of meta-paths on performance

**Fig. 11:**
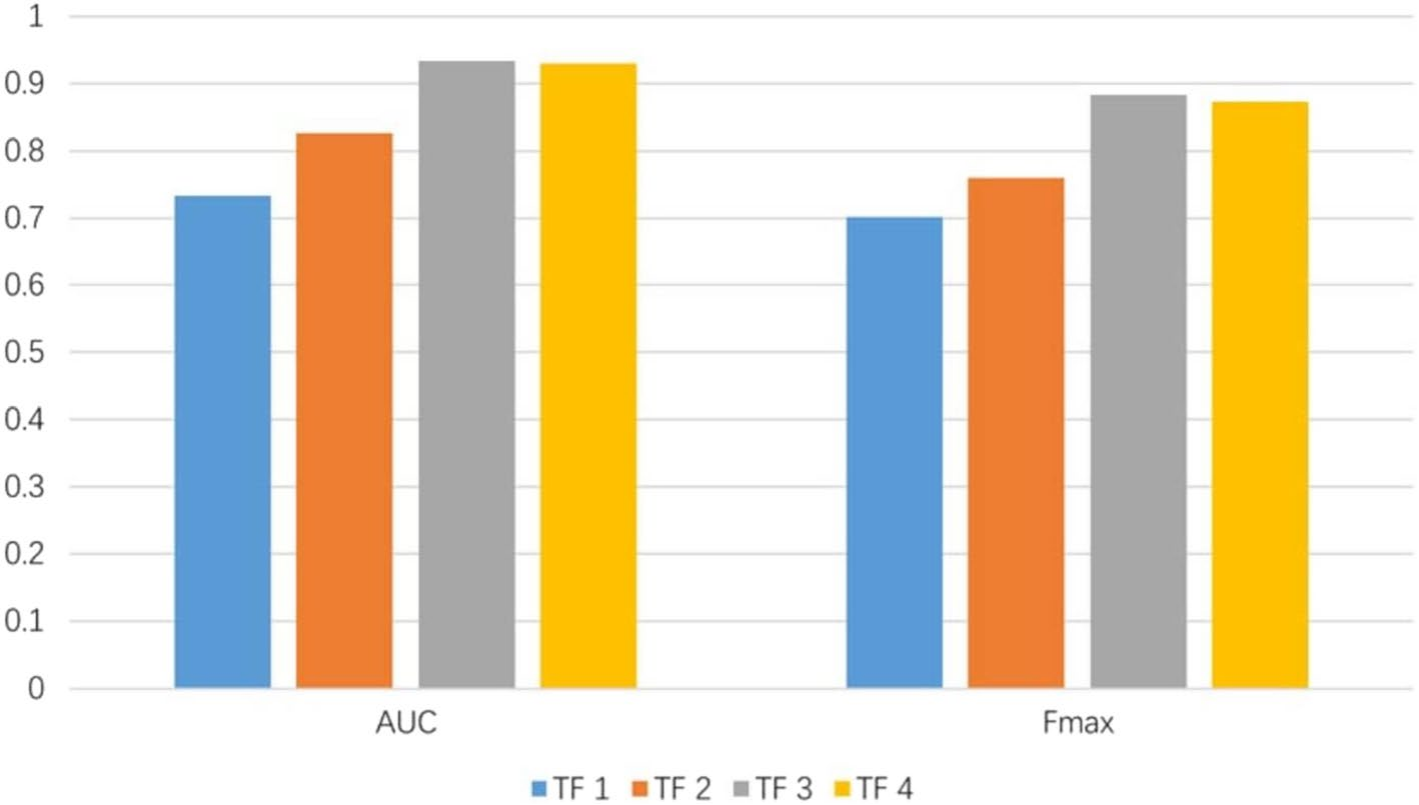
The prediction performance of the XGBOOST model based on different topological features

Generally, the meta-paths with different length make different contributions to the performance. To determine which length can achieve the best performance, we build four sets of topological features (TF1, TF2, TF3 and TF4) by combining the meta-paths with different length respectively. TF1 contains the path counts of meta-path with step size 2 and step size 3 were encoded in 6 commuting matrices. TF2 contains the path counts of meta-path with step size 2, 3 and step size 4 were encoded in 17 commuting matrices. TF3 contains the path counts of meta-path with step size 2, 3, 4 and step size 5 were encoded in 27 commuting matrices. TF4 contains the path counts of meta-path with step size 2, 3, 4, 5 and step size 6 were encoded in 37 commuting matrices. The meta-paths with length 6 are listed in Table 3. Based on the four feature sets, we compare the predictive performance of the classifier. The results are shown in Fig. 11.

**Table 3:** The meta-paths from domains to diseases in the heterogeneous network with length of 6

Pathway scheme	Pathway
DPPDSPS	domain $\rightarrow$ protein $\rightarrow$ protein $\rightarrow$ domain $\rightarrow$ disease $\rightarrow$ protein $\rightarrow$ disease
DPPSPDS	domain $\rightarrow$ protein $\rightarrow$ protein $\rightarrow$ disease $\rightarrow$ protein $\rightarrow$ domain $\rightarrow$ disease
DPPSDSS	domain $\rightarrow$ protein $\rightarrow$ protein $\rightarrow$ disease $\rightarrow$ domain $\rightarrow$ disease $\rightarrow$ disease
DPDSPPS	domain $\rightarrow$ protein $\rightarrow$ domain $\rightarrow$ disease $\rightarrow$ protein $\rightarrow$ protein $\rightarrow$ disease
DPSPPSS	domain $\rightarrow$ protein $\rightarrow$ disease $\rightarrow$ protein $\rightarrow$ protein $\rightarrow$ disease $\rightarrow$ disease
DPSSPDS	domain $\rightarrow$ protein $\rightarrow$ disease $\rightarrow$ disease $\rightarrow$ protein $\rightarrow$ domain $\rightarrow$ disease
DSPDSPS	domain $\rightarrow$ disease $\rightarrow$ protein $\rightarrow$ domain $\rightarrow$ disease $\rightarrow$ protein $\rightarrow$ disease
DSDPSDS	domain $\rightarrow$ disease $\rightarrow$ domain $\rightarrow$ protein $\rightarrow$ disease $\rightarrow$ domain $\rightarrow$ disease
DSSPPSS	domain $\rightarrow$ disease $\rightarrow$ disease $\rightarrow$ protein $\rightarrow$ protein $\rightarrow$ disease $\rightarrow$ disease
DSSPDSS	domain $\rightarrow$ disease $\rightarrow$ disease $\rightarrow$ protein $\rightarrow$ domain $\rightarrow$ disease $\rightarrow$ disease

The demonstration shows that all the evaluation metrics gain the max values on the set of TF3, the worst on TF1. With the number of meta-paths increasing, the prediction performance of the XGBOOST model has a boost. However, the performance of XGBOOST on TF4 is worse than that on TF3. Therefore, we only consider the meta-paths with length less than five in this work.

### Comparison with other machine learning models

**Fig. 12:**
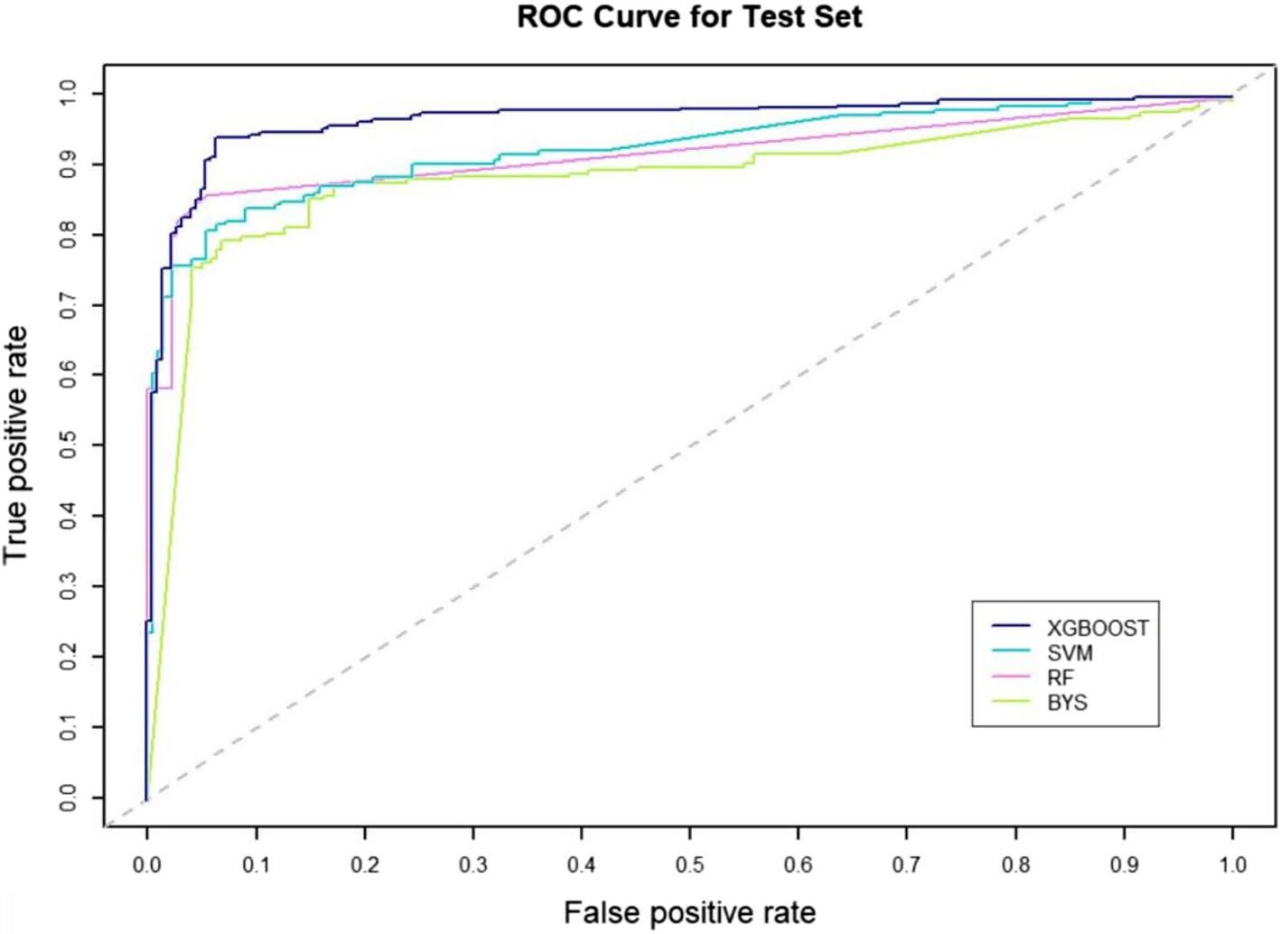
The predictive performances of the different models

To further validate the effectiveness of our chosen XGBOOST model, we compare it with other popular representative machine learning algorithms: RF, SVM and BYS. In order to make a fair comparison of the four methods, we use the same data set for experiments (666 different experimentally validated domain-disease associations as described in the Materials and methods section). The performance is evaluated by AUC, $F_1$ score and the results can be seen in Table 4 and Fig. 12.

**Table 4:** Performance comparison based on different models

Methods	AUC	*F*_1_ scores
XGBOOST	0.9461	0.8942
RF	0.9280	0.8738
SVM	0.9255	0.8718
BYS	0.8805	0.8527

The results show that all models have achieved satisfactory results in predicting the potential associations between structure-based domains and diseases. It can be known that the AUC scores obtained by the above four models are all higher than 0.85 from the table, the $F_1$ scores are all above 0.8. We can find that the XGBOOST model has the best prediction performance with an AUC score of 0.9461, $F_1$ score of 0.8942. The RF model is a little inferior to the XGBOOST model in AUC and $F_1$ score, while the BYS model performs worst with the two metrics. The demonstration indicates that the performance of the XGBOOST model is better than those of other machine learning algorithms. From Fig. 12, we can see that the predictive power of the four models based on different machine learning algorithms follows an order: XGBOOST > RF > SVM > BYS.

## Discussion

Recent studies on the modular nature of human genetic diseases have revealed that diseases share common clinical characteristics are often caused by functionally related genes. It has been becoming important to further locate genes that are responsible for complex diseases, resulting in facilitating the prevention, diagnosis and treatment of these diseases. Notably, a protein typically is composed of several structural domains, each of which is closely related to a specific function of the protein. Therefore, it becomes reasonable to infer associations between protein domains and complex diseases. In this paper, we adopt the ’guilt-by-association’ principle that is based on a global heterogeneous information network consisting of domain-protein association network, protein interaction network, protein-disease association network, disease similarity network, domain-disease association network. Since protein structure is evolutionarily more conserved than sequence, protein function in nature depends on the global architecture, the inner dynamics of folds, and the subtle surface properties that give binding specificity. Hence, we predict the structural-based domains for diseases. According to the global heterogeneous network, we extract meta-path topological features of domain and disease nodes and encode them through commuting matrices. Furtherly, the commuting matrices are normalized by running RW algorithm to improve the predictive performance. To verify this, we build two sets of topological features, namely Feature-I and Feature-II which contains RW normalization of the commuting matrices. Different models (XGBOOST, RF, SVM and BYS) are employed to evaluate the performance on the two feature sets. The predictive performance of each model on Feature-II is improved significantly, which is shown in Table 2. To further investigate the influence of meta-path length on performance, multiple topological feature sets with different meta-path lengths are examined using the XGBOOST model. The results are illustrated in Fig. 11 and we finally utilize the meta-paths with length less than five in this study. Moreover, we compare XGBOOST model with three different baseline models, demonstrating its superior performance on the experimentally validated data set.

Our approach might be extended from the following directions. First, the method enables a new network of biological entities to be integrated into the global network as long as there are connections between the new network and two other networks in the global network. For example, the microRNAs can be integrated into the global network. Second, we only utilize the topological features of the global network. In fact, more features, for example sequence and structural features of domains, could be explored and incorporated into the method.

## Conclusions

With the latest developments in human genetics and computational biology, it has become possible to use the latest technology to identify many of the genes involved in complex diseases. However, narrowing down to a specific structure-based domain can be challenging. At present, structure-based domain-disease association prediction is a relatively new topic, and determining the associations has drawn extensive attention since it is beneficial for drug discovery and personalized treatment of complex diseases. Machine learning methods provide us with a new way to predict the potential associations. Moreover, the semantic network integrating domain knowledge across chemical and biological space can contribute to the research. In this study, we develop a method to build a binary classifier using the topological features based on meta-paths, and predict the potential associations between domains and diseases. Based on its predictive performance in independent test sets, the method is proven to be powerful. We also demonstrate the model based on XGBOOST outperform other machine learning model such as SVM, RF, Naive Bayes and etc. In the future, we will collect more multi-omic data to represent domains and diseases, further infer the associations between them.

## Supplementary information

Additional file 1: The topological feature test set.

Additional file 2: The topological feature test set through RW normalization.

Additional file 3: The topological feature train set.

Additional file 4: The topological feature train set through RW normalization.

## Data Availability

Topological features according to the meta-paths are available online.

## Abbreviations

RF: Random Forest
SVM: Support Vector Machine
BYS: Naive Bayes
XGBOOST: eXtreme Gradient Boosting
